# CDC2-like (CLK) protein kinase inhibition as a novel targeted therapeutic strategy in prostate cancer

**DOI:** 10.1038/s41598-021-86908-6

**Published:** 2021-04-12

**Authors:** Simon Uzor, Sean R. Porazinski, Ling Li, Bethany Clark, Masahiko Ajiro, Kei Iida, Masatoshi Hagiwara, Abdullah A. Alqasem, Claire M. Perks, Ian D. Wilson, Sebastian Oltean, Michael R. Ladomery

**Affiliations:** 1 Faculty of Health and Applied Sciences, University of the West of England, Coldharbour Lane, Bristol, BS16 1QY UK; 2 Institute of Biomedical and Clinical Sciences, Medical School, College of Medicine and Health, University of Exeter, Exeter, EX1 2LU UK; 3 Department of Anatomy and Developmental Biology, Graduate School of Medicine, Kyoto University, Kyoto, Japan; 4 IGFs and Metabolic Endocrinology Group, Bristol Medical School, Translational Health Sciences, University of Bristol, Southmead Hospital, Bristol, BS10 5NB UK; 5 Present Address: Department of Medical Laboratory Science, Ebonyi State University, P.M.B. 53, Abakaliki, Nigeria; 6 Present Address: Garvan Institute of Medical Research, 384 Victoria Street, Darlinghurst, Sydney, NSW 2010 Australia

**Keywords:** Prostate cancer, Alternative splicing, RNA splicing

## Abstract

Dysregulation of alternative splicing is a feature of cancer, both in aetiology and progression. It occurs because of mutations in splice sites or sites that regulate splicing, or because of the altered expression and activity of splice factors and of splice factor kinases that regulate splice factor activity. Recently the CDC2-like kinases (CLKs) have attracted attention due to their increasing involvement in cancer. We measured the effect of the CLK inhibitor, the benzothiazole TG003, on two prostate cancer cell lines. TG003 reduced cell proliferation and increased apoptosis in PC3 and DU145 cells. Conversely, the overexpression of CLK1 in PC3 cells prevented TG003 from reducing cell proliferation. TG003 slowed scratch closure and reduced cell migration and invasion in a transwell assay. TG003 decisively inhibited the growth of a PC3 cell line xenograft in nude mice. We performed a transcriptomic analysis of cells treated with TG003. We report widespread and consistent changes in alternative splicing of cancer-associated genes including *CENPE*, *ESCO2*, *CKAP2*, *MELK*, *ASPH* and *CD164* in both HeLa and PC3 cells. Together these findings suggest that targeting CLKs will provide novel therapeutic opportunities in prostate cancer.

## Introduction

The vast majority of human genes are alternatively spliced (> 94%), expressing splice isoforms that often exhibit antagonistic properties^1,2^. Aberrant alternative splicing contributes to the pathogenesis and progression of cancer^3^ and plays an essential role in all hallmarks of cancer^4^. Dysregulated alternative splicing has even been proposed as new a cancer hallmark^5^, and is associated with the effectiveness of treatments and the development of drug resistance^6^.There are multiple mechanisms through which the expression of oncogenic splice isoforms can increase. Splice sites can be mutated directly; cryptic splice sites can be activated, and regulatory sequences known as exonic or intronic enhancers or silencers can be disrupted. Mutations that affect the pre-mRNA splicing machinery are also associated with cancer. SF3b is a heptameric complex and component of the U2 snRNP that contacts the intron’s branch site during splicing. Mutations of SF3b are associated with several cancers including leukemia and melanoma, and cause disruption to the normal recognition of branch sites^7^. The expression of alternative splicing regulators can also be altered in cancer, including splice factor proteins and noncoding RNAs that regulate alternative splicing. SRSF1 is a well-studied RNA-binding splice factor, and the first to be described as a proto-oncogene^8–10^. As well as splice factors, it is now clear that splice factor protein kinases can also contribute to the oncogenic phenotype. These include the SRPKs (SR protein kinases), CLKs (CDC2-like protein kinases), DYRK (dual-specificity tyrosine regulated kinase) and PRP4K (pre-mRNA splicing 4 kinase) families. Splice factor kinases are integrated within established cell signalling networks and modify the localisation and activity of splice factors and spliceosome components. They have key roles in tumorigenesis and their activity is associated with response to treatment. We recently demonstrated that inhibition of the splice factor kinase SRPK1 with the compound SPHINX reduces prostate cancer xenograft growth through an anti-angiogenic alteration of VEGFA splicing^11^. Here we have turned our attention to another family of splice factor kinases, the CLKs (CDC2-like).

The human *CLK1* gene was first described in 1991^12^. It encodes a 484 amino-acid dual specificity protein kinase with the characteristic signature LAMMER sequence in the C-terminal catalytic domain^13^. The first 130 amino-acids consist of a regulatory region required for CLK1 to interact with substrates, including the oncogenic splice factor SRSF1. There are four CLK genes in humans, *CLK1-4*. CLK1, 2 and 4 are ubiquitously expressed, whereas CLK3 expression is particularly high in spermatozoa^14^. The role of CLK3 in human spermatogenesis has not yet been elucidated; interestingly, a CLK-like gene prevents germ cell over-proliferation in *Drosophila melanogaster*^15^.

The developmental and pathophysiological roles of the four members of the human CLK family are complex. CLK1 is involved in adipogenesis where its activity is regulated through Akt-mediated phosphorylation^16^. CLK2 is required for the control of diet-induced thermogenesis in brown adipose tissue^17^ and suppresses hepatic gluconeogenesis^18^. Exercise reduces CLK2 expression in the liver of obese mice, preventing fat build up^19^. CLK2 inhibition may provide benefit for the treatment of Phelan-McDermid Syndrome^20,21^, a complex inherited disorder caused by deletion of the *SHANK3* gene in which affected children experience hypotonia, developmental delay, autism and occasional seizures. CLK2 is involved in the regulation of *tau* alternative splicing, and the expression of an inactive isoform of CLK2 is elevated in affected brain areas of sporadic Alzheimer’s patients^22^. CLK1 inhibition may benefit the treatment of Duchenne’s muscular dystrophy as its inhibition promotes the skipping of a mutated dystrophin exon^23^. The inhibition of CLKs with small molecule inhibitors could also provide avenues for the treatment of infection. To this end, CLK1 inhibitors show promise for the treatment of influenza^24^, HIV-1^25^ and *Plasmodium falciparum* infection in malaria^26^.

Increasing evidence now links the CLKs to cancer. The *CLK2* gene is amplified in a significant proportion of breast tumours and its downregulation inhibits cancer cell growth in cell culture and xenografts. Loss of CLK2 in luminal breast cancer cells leads to the expression of mesenchymal splice variants, such as the skipping of exon 11a of *ENAH*, encoding an actin cytoskeleton protein^27^. The targeting of CLK2 with the inhibitor T-025 in an allograft model of MYC-driven spontaneous breast cancer resulted in significant anti-tumour effects, including the induction of skipped exons, of apoptosis, and growth suppression^28^. CLK1 and CLK2 inhibition may provide benefit in the treatment of triple-negative breast cancer (TNBC). The compound CC-671, an inhibitor of both CLK2 and the dual-specificity protein kinase TTK, blocked the growth of two cell lines and a patient-derived TNBC xenograft^29^. CLK2 also affects cell survival following ionizing radiation. The overexpression of CLK2 inhibits cell death induced by doses of two Gray; this effect is dependent on its phosphorylation by Akt^30^.

We previously showed that targeting SRPK splice factor kinases in prostate cancer cells could could provide a novel therapeutic strategy ^11^. Here we have targeted CLKs in prostate cancer cells with the benzothiazole TG003, a well-established small molecule inhibitor of CLK1 and CLK4^31^. We find that TG003 has potent anti-tumour properties in prostate cancer cells in vitro and in a xenograft model, and that it causes significant changes in the alternative splicing of cancer-associated genes.

## Materials and methods

### Cell culture and reagents

PC3 and PNT2 cells were purchased from ECACC and DU145 cells from ATCC. Cell lines were grown in Dulbecco’s Modified Eagle’s Medium (DMEM), plus 2 mM glutamine and 10% fetal bovine serum (FBS). The benzothiazole CLK inhibitor TG003 was dissolved in dimethyl sulfoxide (DMSO). Unless otherwise specified, cell media and chemicals were purchased from Sigma-Aldrich.

### Cell proliferation assays

To count live cells, 3 × 10^5^ cells were seeded into six-well plates containing 2 ml of cell culture medium and treated with 1, 10 and 50 µM TG003. Every 24 h cells were trypsinised and counted in a haemocytometer (Neubauer chamber). Trypan blue (0.4% w/v) was used as an exclusion stain.

To measure cell proliferation, 3 × 10^5^ cells were seeded into six-well plates containing coverslips and serum-starved in 4 ml of DMEM for eight hours, then replaced with full medium. Cells were treated with 1 µM, 10 µM and 50 µM TG003. After 48 h incubation, media was removed and cells were washed twice. Washed cells were fixed in 4% (w/v) paraformaldehyde (PFA) for ten minutes and permeabilized in 0.25% (v/v) triton in PBS.

Fixed cells were blocked in 10% FBS-PBS for one hour and incubated overnight at 4 °C with 1:1000 anti-Ki67 primary antibody (Abcam, Cambridge, UK). Cells were washed three times in PBS before incubation for one hour at room temperature with secondary antibody, AlexaFluor 488 goat anti-rabbit (Molecular Probes), diluted 1:500, followed by washing. Cells were then counterstained with DAPI for four minutes and further washed twice before mounting. Images were obtained with an Image Pro Plus (TE 300 Nikon Japan) microscope and the percentage proliferation calculated with ImageJ software.

### Apoptosis assays

3 × 10^5^ cells were seeded in six well plates and treated for up to 72 h with 1 µM, 10 µM and 50 µM TG003. Media was removed and replaced with caspase-3/7 green detection reagent (Thermofisher Scientific, as per manufacturer instructions) and placed in a 37 °C incubator for 45 min. Images were obtained at Fluor 488 green channel with an Image Pro Plus microscope. The percentage of caspase 3/7 positive cells was determined using ImageJ software.

### Scratch assays

A pipette tip guided by a clean glass slide was used to make a straight-line scratch in a confluent prostate cancer cell line lawn in six well plates. Floating scratched cells were gently removed. A Lumascope (Labtech International, USA) was used to record images for 2 h. ImageJ was used to analyse the extent of scratch closure in both controls and TG003-treated cells.

### Transwell cell migration and invasion assays

For cell invasion measurements, PC3 cells were serum-starved for eight hours and subsequently treated for 48 h with TG003. Matrigel (Sigma-Aldrich, 7.9 mg/ml) was diluted with serum-free DMEM and 100 µl of the mixture was added into transwell inserts in 24-well plates. Matrigel was solidified overnight at 37 °C and then 500 µl of DMEM containing 10% FBS was added to the lower chamber as chemoattractant, while 300 µl of serum-free DMEM containing 1.0 × 10^5^cells was added to the top chamber and plates were incubated for 24 h at 37 °C. The media was removed and cells were washed twice in PBS and subsequently fixed in 4% (w/v) PFA at room temperature for ten minutes. Non-invasive cells inside the insert were removed with a cotton swab. Cells were permeabilized in absolute methanol for 20 min at room temperature after being washed twice in PBS. The insert was cut and inverted onto a glass slide and incubated with Hoechst (ThermoFisher Scientific) for 1 h. Invasive cells were counted using an Image Pro Plus microscope. For the cell migration assay the same method was used, but inserts were not coated with matrigel.

### siRNA knockdown of CLK1

5 × 10^5^ PC3 cells were seeded in six well plates with antibiotic-free growth medium and allowed to settle overnight. siRNA (control (sc-37007) and CLK1 (sc-60404), Santa Cruz USA) and lipofectamine (RNAiMAX Fisher Scientific) were mixed in Optimem medium as per manufacturer instructions. siRNA was added to cells topped up with 1 ml Optimem (Thermofisher Scientific) and incubated for 4 h at 37 °C. Next 2 ml of antibiotic-free DMEM was added and cells grown for 48 h. Cells were lysed with standard RIPA buffer including a protease inhibitor cocktail. Proteins were quantified using a standard Bradford assay and examined via SDS PAGE.

### Immunofluorescence assay

3.0 × 10^5^cells were seeded and treated with varying concentrations of TG003 on clean cover slips in six well plates. Cells were washed three times with warm PBS and fixed in 4% (w/v) PFA. Cells were washed three times again, permeabilized in 0.25% (v/v) triton-PBS for 10 min and blocked in 10% FBS/PBS for an hour. E-cadherin (24E10 rabbit polyclonal. Cell Signalling Technology) and vimentin (sc-6260 mouse monoclonal, Santa Cruz, USA) primary antibodies were diluted 1:150 and 1:400 respectively in 10% FBS-PBS and incubated overnight at 4 °C. After PBS washes, donkey anti-rabbit AlexaFluor-568 for E-cadherin (1:750, Molecular Probes) and anti-mouse AlexaFluor-488 for vimentin (1:1000, Molecular Probes) were applied in 10% FBS-PBS at room temperature for one hour. Cells were washed with PBS and counterstained in Hoechst (ThermoFisher Scientific) for 10 min, followed by mounting of coverslips**.** Images were obtained using fluorescence microscopy (Image ProPlus, TE 300 Nikon Japan) using a 60 × objective and analysed with ImageJ software.

### Generation of stable CLK1 overexpressing cells

PC3 cells were transfected using FuGene® HD Transfection Reagent (Promega). 3 µg of pAM92 CLK1-expressing plasmid provided by Prof. Masatoshi Hagiwara, or empty vector (p3xFLAG-CMV-14, Sigma-Aldrich) were diluted in 100 µl Opti-MEM I reduced serum medium (Thermofisher, Gibco). 6 µl of the FuGene® HD Transfection Reagent was pipetted directly into the medium containing diluted plasmid to form the transfection complex, and then the complex was mixed and incubated at room temperature for 15 min. The transfection complex was added to cells and the wells were swirled to ensure even distribution. The cells were incubated for 72 h before splitting to T25 flasks. Stable transfectants were selected using 500 µg/ml Geneticin (Thermofisher, Gibco) and CLK1 overexpression verified by western blotting.

An Alamar Blue (resaruzin, ThermoFisher) proliferation assay was performed on the stably transfected cells, following the manufacture’s protocol. PC3, PC3 EV or PC3 CLK1 cells were seeded in 96-well plate at 10,000 cells per well and then treated with either control (DMSO) or 10uM TG003 the next day. After 48 h treatment, medium was refreshed with serum free media containing 10% Alamar Blue in each well and incubated at 37℃, including no-cell control wells as blank. Fluorescence (excitation of 560 nm, and emission at 590 nm) was measured at three hours in each well using a SpectraMax M2e Plate Reader (Molecular Devices). The proliferation rate was calculated as following: percentage difference between treated and control cells = FI 590 of TG003/FI 590 of control *100. The data were analysed by Prism using one-way ANOVA. Six repeats were applied for each treatment.

### Xenograft analysis

One million PC3 cells were subcutaneously injected into each right flank of CD1- nude mice, 12 mice in total (two month old mice, obtained from Charles River Laboratories). Tumour diameters were measured by caliper two times per week. TG003 (corresponding to 50 µM final concentration in the mouse) and vehicle control were injected intraperitoneally two times a week when tumour size reached 3 mm × 3 mm, 6 mice per treatment group. Mice were culled when control tumours size reached 12 mm in one direction and tumours were extracted, imaged and weighed. Tumour volumes were calculated using the formula ‘volume = [(length + width)/2] × length × width’. Quantitation of the tumour volumes were analysed by Two-way ANOVA using Prism 6 (GraphPad Software, Inc.). All animal experiments were conducted in accordance with UK legislation and with local ethical committee approval (Animal Welfare & Ethical Review Board- University of Exeter). We confirm that all animal experiments were carried out in compliance with the ARRIVE guidelines.

### Western blotting

Determination of protein concentration was performed using a Bradford assay as per manufacturer’s instruction. A standard protein concentration curve was generated using Fluostar Optima, BUG Labtech, UK. 20 µg protein samples were prepared in standard Laemmli buffer (at a 1:1 ratio). Protein samples were separated using standard SDS-PAGE 10% acrylamide gels. Wet transfer to a PVDF membrane was performed in standard ice-cold transfer buffer, transferring for 2 h at 50 V. Membranes were blocked in 5% bovine serum albumin and probed overnight with 1:5000 primary antibodies, CLK1 (Sigma-Aldrich R1471-1S) and pan-phospho SR protein (mAb1H4, Insight Biotech. sc-13509). After washing three times in TBST buffer, suitable secondary antibodies (1:15,000 Licor anti-rabbit) were applied (two hours at room temperature), followed by TBST washing. Following chemiluminescence (Luminata forte, Millipore Ltd), membranes were imaged using an Odyssey imaging platform (LI-COR, USA).

### mRNA sequencing (RNA-Seq) analysis

RNA-Seq data from SRA ID: SRP075278 (SRR3616963, SRR3616964, SRR3616967, and SRR3616968)^32^ were used for the analysis. Sequence reads with average Phred quality scores under 17, or derived from rRNA, tRNA, snRNA, or snoRNA, and those from repetitive elements, identified in Repbase database^33^, were omitted from the dataset. Quality-controlled mRNA-seq reads were mapped to the human genome sequences (hg19) with STAR (ver. 2.4.1d)^34^, and analyzed with rMATS (ver. 4.0.2)^35^. Ensembl annotation ver. GRCh37.75 was used for the calculation^36^. Following the calculation, alternative splicing (AS) events which passed the following criteria were processed as significant AS events; FDR < 0.1, related reads ≥ 10 in either of splicing forms, |dPSI|≥ 0.05. We then listed splicing events which affect “productive forms”, defined as an alternative mRNA isoform providing CDS for intact protein products^37^. Splice isoforms that encode translational products in the UniProt database were sorted out as productive forms. Gene ontology (GO) analysis for TG003-susceptible events was conducted using metascape server^38^.

### Reverse transcription-coupled polymerase chain reaction (RT-PCR)

HeLa and PC3 cells were treated with DMSO (0.1%) or TG003 (20 µM) for 24 h, and then total RNA was extracted using direct-zol RNA miniprep kit (Zymo Research, Irvine, CA, USA), and reverse transcription (RT) was performed with Prime Script RTase (Takara Bio Inc., Shiga, Japan). RT products were then applied for PCR with ExTaq DNA polymerase (Takara Bio Inc.). Primers used for RT-PCR were as follows: oAM441 (5′-GGAACTAAAAACTGCTCGTATGC-3′) and oAM442 (5′-CTCAGGCTTTCCGTAAGGTG-3′) for CENPE exon 38 splicing, oAM421 (5′-GTGGACAAGCCAGTGCTACC-3′) and oAM422 (5′-ACAGCAGACTTAGGAGTATATAC-3′) for MELK exon 13 splicing, oAM423 (5′-AGAACCCCAGAATATCGAAG-3′) and oAM424 (5′-ACGTGGTAACTATGCTCGGT-3′) for ASPH exon 6 splicing, oAM425 (5′-ACCGAGCATAGTTACCACGT-3′) and oAM426 (5′-CTACAATTACCTGTGAATCT-3′) for ASPH exon 8 splicing, oAM427 (5′-AGTTAGTGATTGTCAAGTGG-3′) and oAM428 (5′-AATGAAACTGGCTGCATCAAAG-3′) for CD164 exon 5 splicing, oAM429 (5′-TCGTGTTGGTTCTGCCACAT-3′) and oAM430 (5′-ACCAGTCGTCTTGCAATGCG-3′) for ESCO2 exon 9 splicing, oAM433 (5′-GCAGTCCGCATTCAAAGAGC-3′) and oAM434 (5′-TTTATGTGTGTCAATTACTACAG-3′) for CKAP2 exon 3 splicing, and oAM13 (5′-CCAACCGCGAGAAGATGACC-3′) and oAM14 (5′-AGCTTCTCCTTAATGTCACG-3′) for ACTB. Percent spliced-in (PSI) for RT-PCR products was calculated by (inclusion product)/(inclusion product + skipping product)), according to densitometric intensities normalized by individual molecular weights.

## Results

### The benzothiazole CLK inhibitor TG003 reduces cell proliferation and induces apoptosis

**Figure 1:**
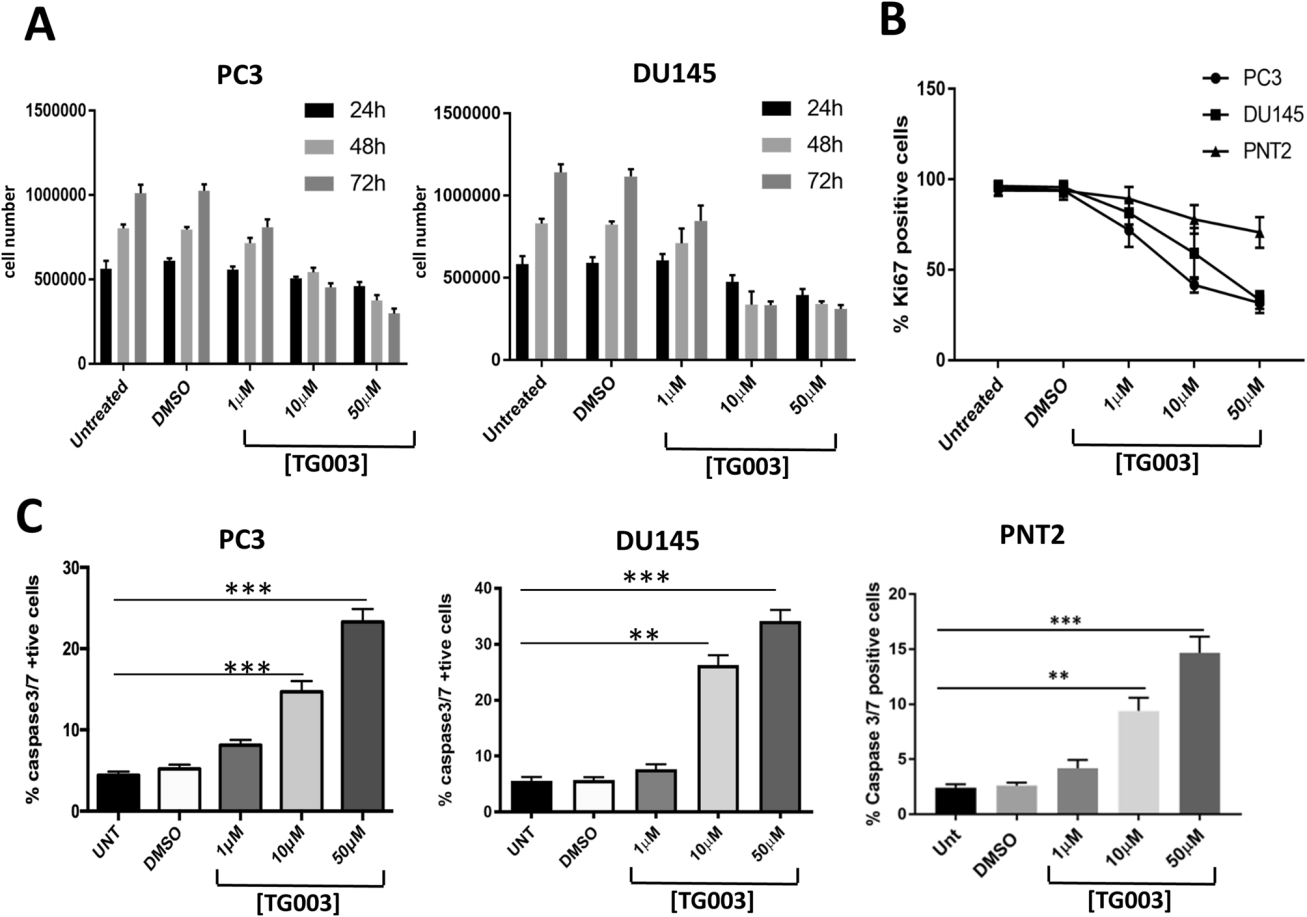
Effect of TG003 on cell proliferation and apoptosis. (**A**) PC3 and DU145 prostate cancer cell lines were treated with 1, 10 and 50 µM TG003. Viable cells were counted at 24, 48, and 72 h following treatment (trypan blue assay). Untreated and DMSO controls are included. (**B**) The proportion of Ki67-positive proliferating cells was measured in PC3 and DU145 prostate cancer and in PNT2 normal prostate epithelium cells. (**C**) The percentage of apoptotic cells was measured via caspase 3/7 staining following 48 h of exposure to TG003. N = 3 repeats in all experiments. **p < 0.01; ***p < 0.001.

We selected the benzothiazole TG003, a well-characterised CLK inhibitor. TG003 was originally developed in Prof. Masatoshi Hagiwara’s laboratory and first described in 2004^31^. TG003 is a small molecule inhibitor, with low nanomolar IC_50_ values for CLK1 and its close homologue CLK4 (15-20 nM)^31^. We treated PC3 (established from a bone metastasis of a grade IV prostate cancer) and DU145 cells (derived from a CNS metastasis of a prostate adenocarcinoma), both hormone-independent cell lines, and for comparison PNT2 immortalised normal prostate epithelium cells with a single dose of 1 µM, 10 µM and 50 µM TG003 for up to 72 h. We confirmed the previously reported ability of TG003 to reduce SR protein phosphorylation^31^ (Supplementary Figure S1A). In both prostate cancer cell lines 1 µM TG003 reduced cell proliferation. The effect was more evident at 10 and 50 µM where cells continued to divide in the initial 24 h but then declined in number markedly (Fig. 1A). Next, we measured the cell proliferation marker Ki67 following 48-h exposure to TG003 with the same range of concentrations (Fig. 1B). The percentage of Ki67-positive declined with increasing TG003 concentrations. At 50 µM TG003, only a third of cells were Ki67-positive. The normal prostate epithelium cell line PNT2 appeared less sensitive to TG003, as the percentage of Ki67-positive cells declined at a slower rate.

Previous reports suggest that inhibitors of CLKs and SPRKs can increase apoptosis^39^. We therefore investigated the effect of TG003 on apoptosis by measuring activated caspase 3/7 48 h after a single TG003 dose. In all three cell lines TG003 caused a noticeable increase in apoptosis, albeit less markedly in the PNT2 cells (Fig. 1C).

### TG003 reduces cell migration and invasion

**Figure 2:**
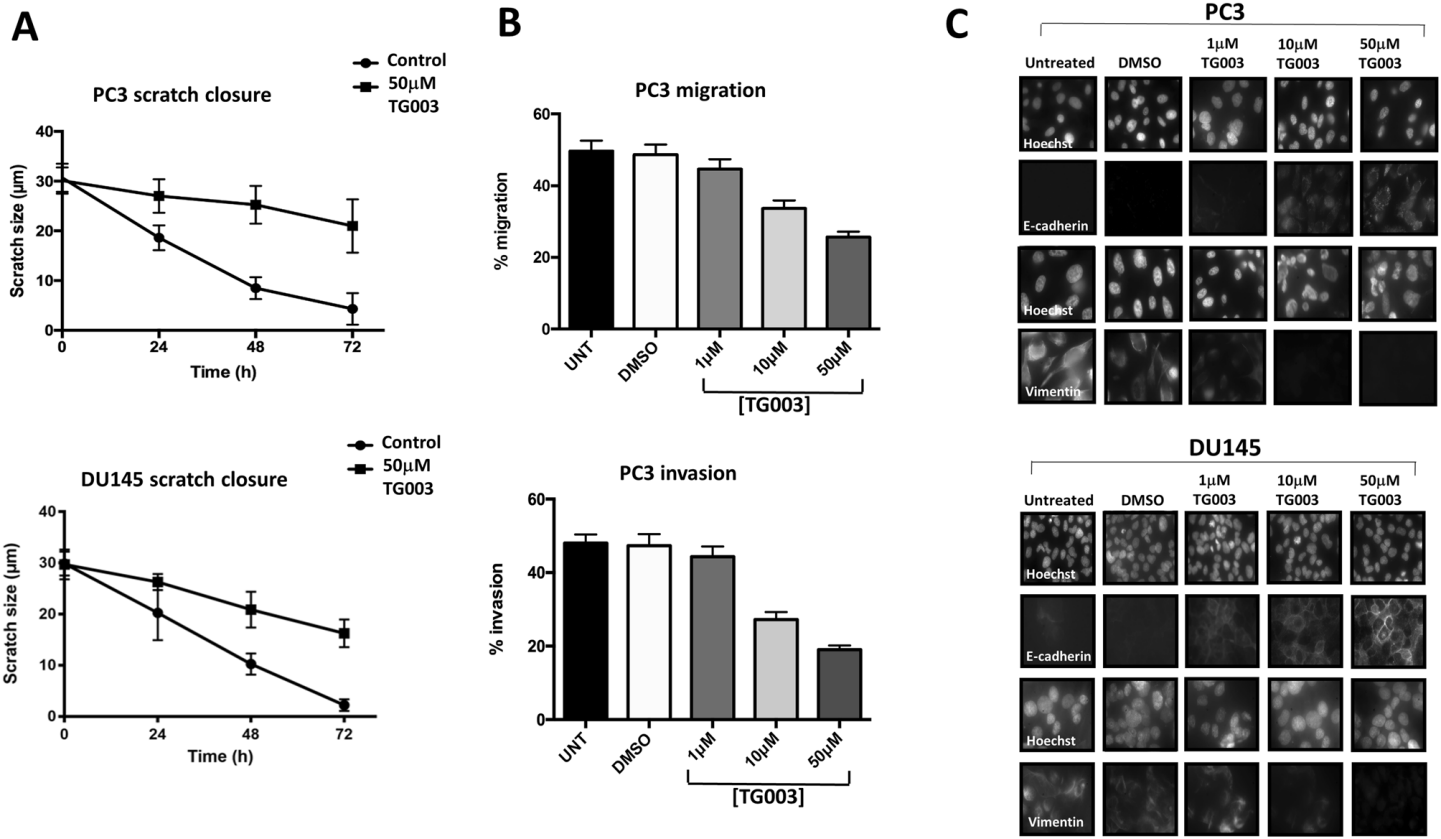
Effect of TG003 on scratch closure, cell migration and invasion. (**A**) 30 µm scratches were applied to confluent cell monolayers and allowed to close over 72 h. PC3 and DU145 cells were treated with 50 µM TG003. (**B**) Cell migration and invasion were measured using a transwell assay following treatment of PC3 cells with 1, 10 and 50 µM TG003. (**C**) Following treatment of PC3 and DU145 cells with 1, 10 and 50 µM TG003, E-cadherin and vimentin expression was determined by immunohistochemistry; with Hoechst counterstain. N = 3 repeats in all experiments. *p < 0.05; **p < 0.01; ***p < 0.001; ****p < 0.0001.

Previous reports suggest that CLKs can influence cell migration and invasion. CLK2 inhibition changes the expression and alternative splicing of EMT-associated genes in breast cancer^27^, and the activation of the splice factor SPF45 following its phosphorylation by CLK1 promotes cancer cell line migration and invasion of ovarian cancer cells^40^. We examined the effect of TG003 on the ability of cells to close a 30 µm gap (scratch assay, Fig. 2A). The gap was closed after 72 h by both PC3 and DU145 cells, but this was substantially slowed down by 50 µM TG003. Treatment with 10 µM TG003 resulted in an intermediate effect (data not shown). We then used a transwell (Boyden) assay to measure cell migration and cell invasion. Both migration and invasion of PC3 cells was significantly reduced by 10 µM of TG003, with a more marked effect at 50 µM TG003 (Fig. 2B). Similar results were obtained with DU145 cells (data not shown). These results suggest that CLK activity helps to promote a metastatic phenotype. Consistent with this hypothesis, we examined the expression of E-cadherin (associated with an epithelial phenotype) and vimentin (associated with a mesenchymal phenotype). In both PC3 and DU145 cells we observed that TG003 caused a clear upregulation of E-cadherin in parallel with a reduction in vimentin expression (Fig. 2C).

### Knockdown of CLK1 confirms the effect of TG003

**Figure 3:**
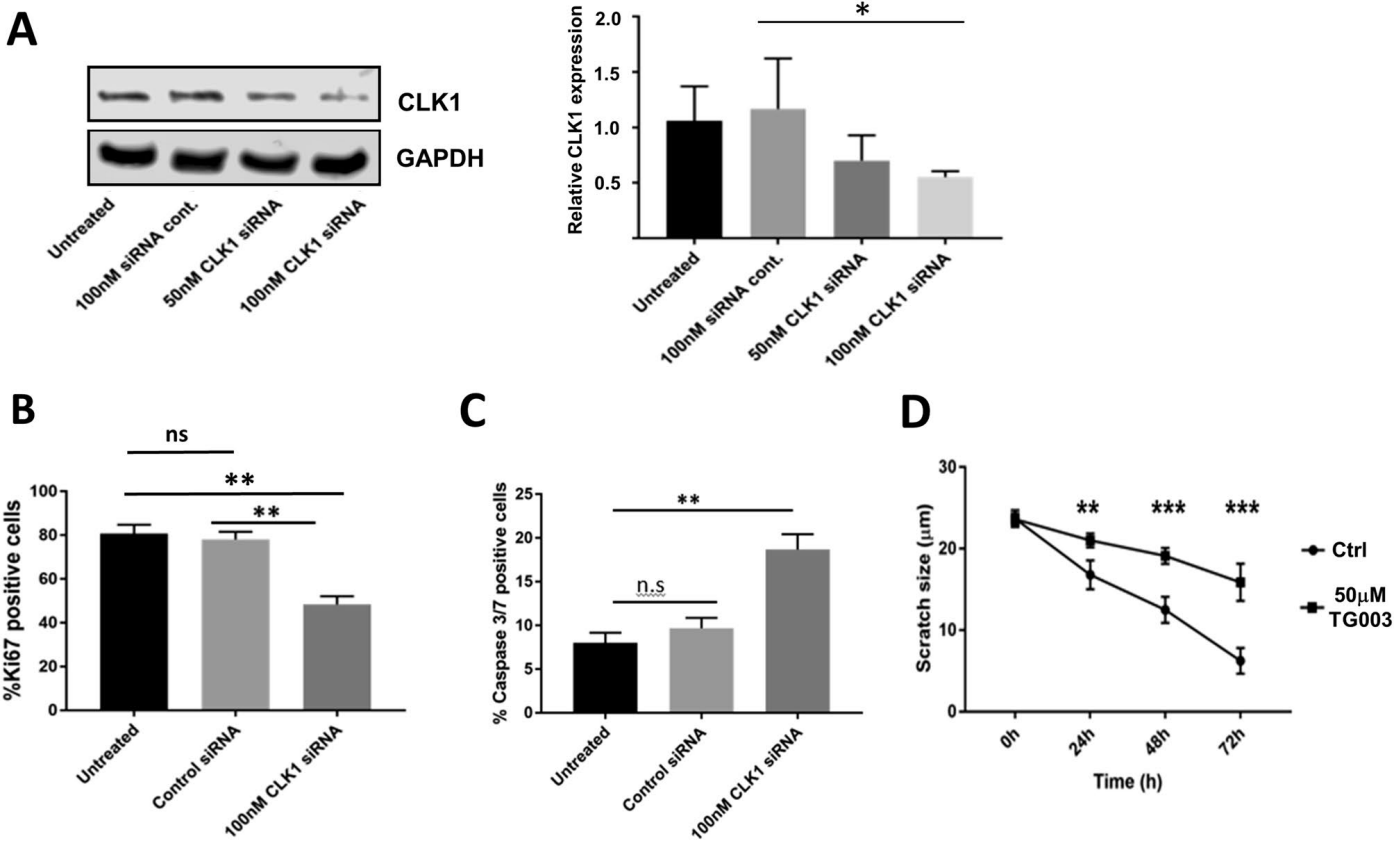
CLK1 knockdown confirms the effect of TG003. **(A**) siRNA directed against CLK1 was transfected into PC3 cells. CLK1 and GAPDH signals are shown. The knockdown was quantified from N = 3 repeats (right panel). The effect of CLK1 knockdown was determined on cell proliferation via Ki67 staining (**B**); apoptosis with caspase 3/7 staining (**C**) and on scratch closure (**D**). N = 3 repeats in all experiments. ns, *p* > 0.05; *p < 0.05; **p < 0.01; ***p < 0.001.

The benzothiazole TG003 is most effective against CLK1 and CLK4^31^; however, like other kinase inhibitors, it exhibits cross-reactivity with other kinases including CLK2 and DYRK1A. In order to confirm that the effect of TG003 on proliferation, apoptosis and cell migration was due to CLK inhibition we knocked down CLK1 in PC3 cells using siRNA (Fig. 3A). We observed that CLK1 knockdown significantly reduced the number of Ki67-positive cells (Fig. 3B) and doubled the percentage of apoptotic cells (Fig. 3C). We performed a scratch closure assay and observed that CLK1 knockdown significantly reduced the rate of scratch closure (Fig. 3D). In summary, the knockdown of CLK1 had the same effect as TG003.

**Figure 4:**
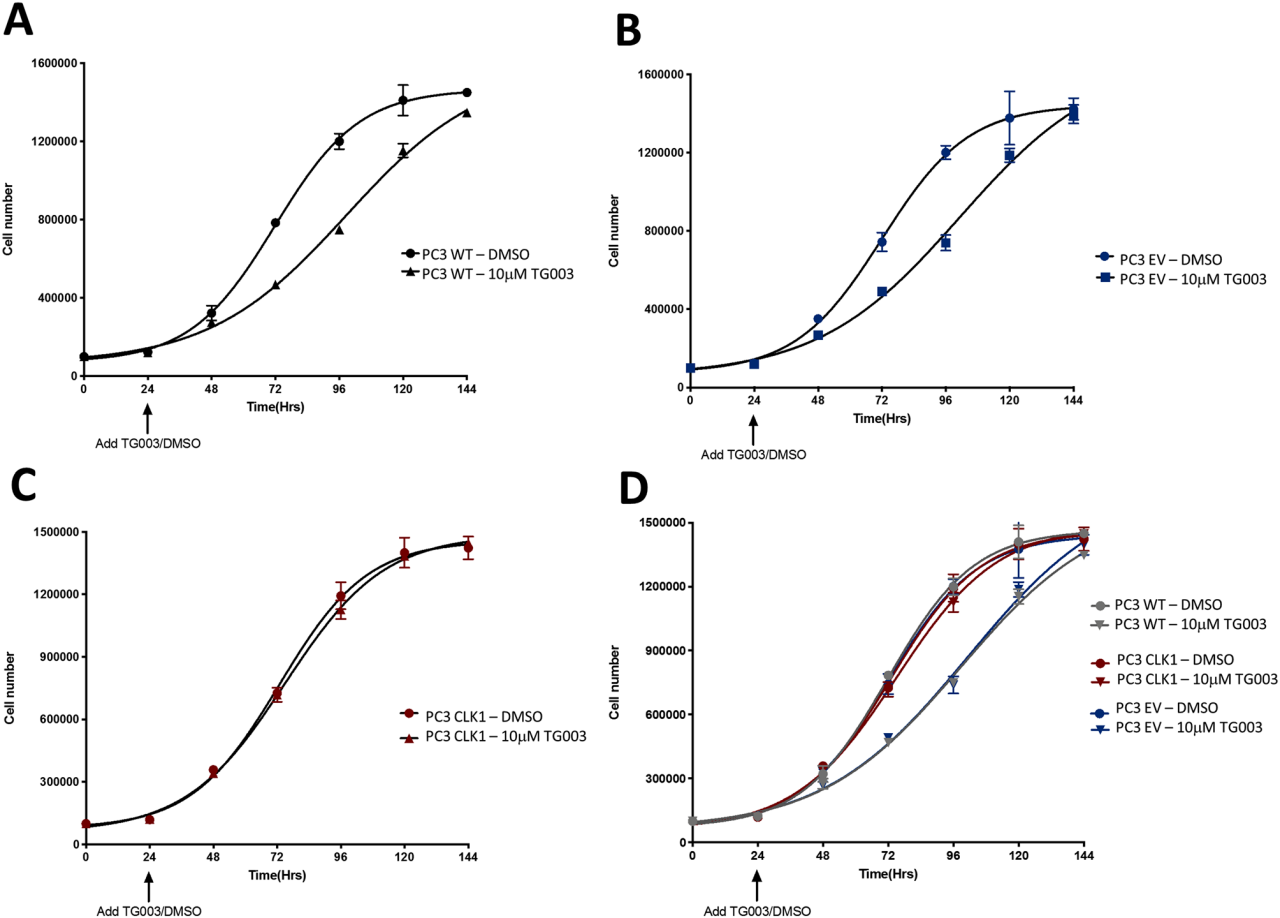
Stable CLK1 overexpression reduces TG003 sensitivity. 10,000 cells were seeded in 96-well plates and a single dose of 10 µM TG003 added after 24 h. Cells were counted every 24 up to 144 h (Alamar Blue assay); DMSO alone and TG003-treated. (**A**) Wild-type (WT) cells; (**B**) PC3 cells stably transfected with empty vector (EV); (**C**) PC3 cells stably transfected with a CLK1-expressing vector; (**D**) combination of the three. N = 3, ****p < 0.001, two-way ANOVA.

To provide additional confirmation that TG003′s effect on cell proliferation is due to CLK1 inhibition, we generated stable CLK1-overexpressing PC3 cells (Supplementary Fig. S1B) and measured sensitivity to TG003. PC3 cells stably transfected with the empty vector (EV) and with a CLK1-expressing vector were seeded into 96-well plates (10,000 cells/well). After 24 h, cells were treated with 10 µM TG003. We counted cell numbers for five days following TG003 treatment. The rate of increase in cell numbers was significantly diminished in both TG003-treated parental and empty vector (EV) PC3 cells, whereas there was no difference between untreated and TG003-treated CLK1-overexpressing cells (Fig. 4). These results indicate that the overexpression of CLK1 renders PC3 cells less sensitive to TG003.

### Inhibition of xenograft growth by TG003

**Figure 5:**
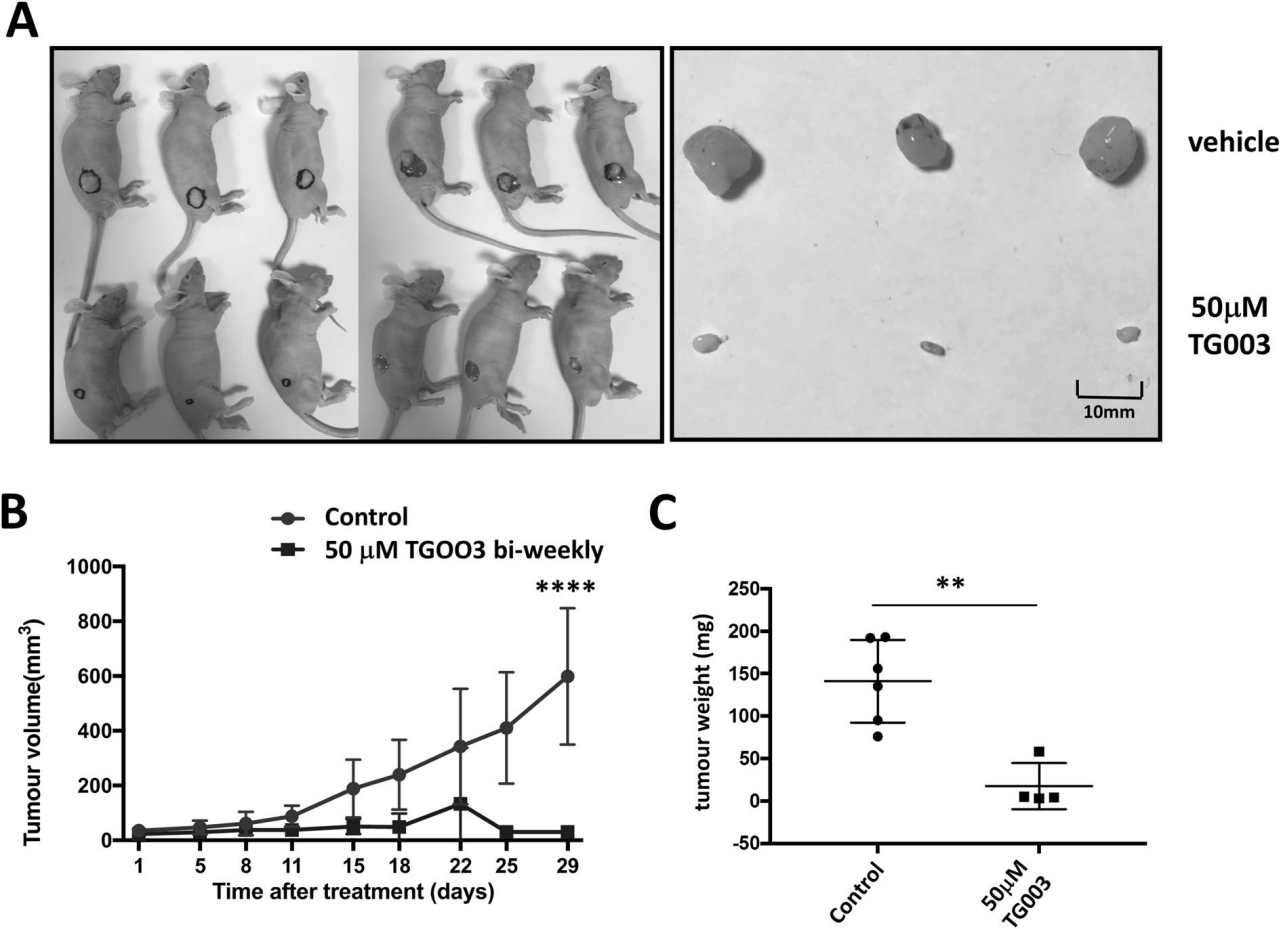
Effect of TG003 on xenograft growth**.** One million PC3 cells were injected subcutaneously into cohorts of six CD1-nude mice and tumour volumes measured over a period of 29 days, comparing vehicle control versus 50 µM TG003. (**A**) Image of the excised tumours; (**B**) tumour volumes measured in in mm^3^ and (**C**) tumour weight in mg. Tumour sizes were statistically different, two-way ANOVA. **p < 0.01; ****p < 0.0001.

Having established that TG003 has a strong effect on PC3 proliferation in vitro, we tested the effectiveness of TG003 in vivo on PC3 xenografts. PC3 cells grow vigorously, are highly invasive and well suited for xenograft experiments^41^. We injected one million cells subcutaneously into the flanks of nude mice. When tumours reached 3 × 3 mm in diameter, a solution of TG003 calculated to give 50 µM final concentration in the mouse was injected intraperitoneally twice a week. After 29 days the untreated tumours reached the maximum permitted size of 12 mm and the experiment was terminated. The TG003 treatments clearly prevented the xenografts growing and the volumes of the tumours in treated animals did not increase (Fig. 5). Other than the clear inhibition of tumour growth, the injection of mice with TG003 bi-weekly did not cause any obvious adverse effects.

### RNA-Seq analysis of the effect of TG003 treatments

**Figure 6:**
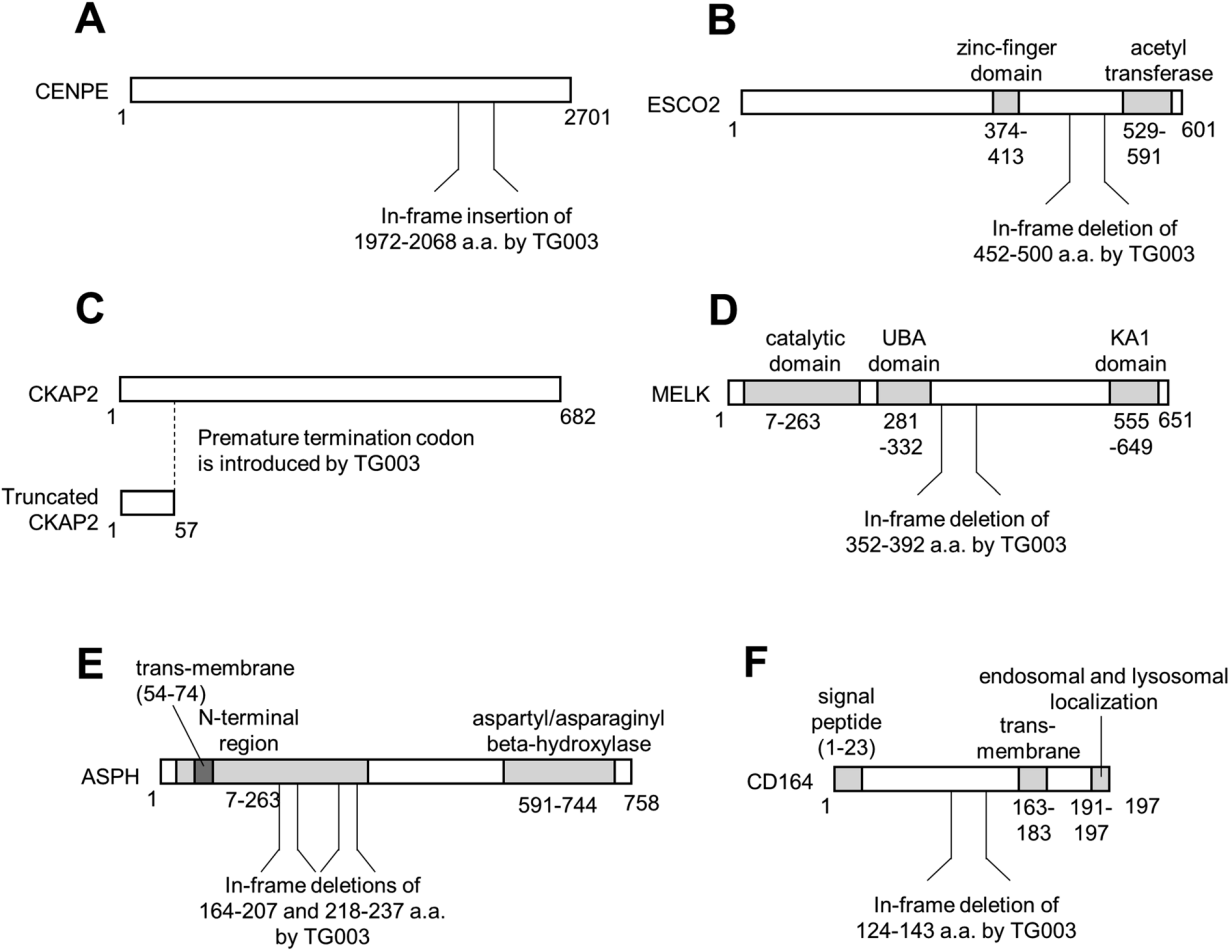
Effect of TG003 on protein structure caused by alternative splicing events. Diagrams of protein structures are shown, indicating the effect of the alternative splicing events detailed in Table 1.

**Figure 7:**
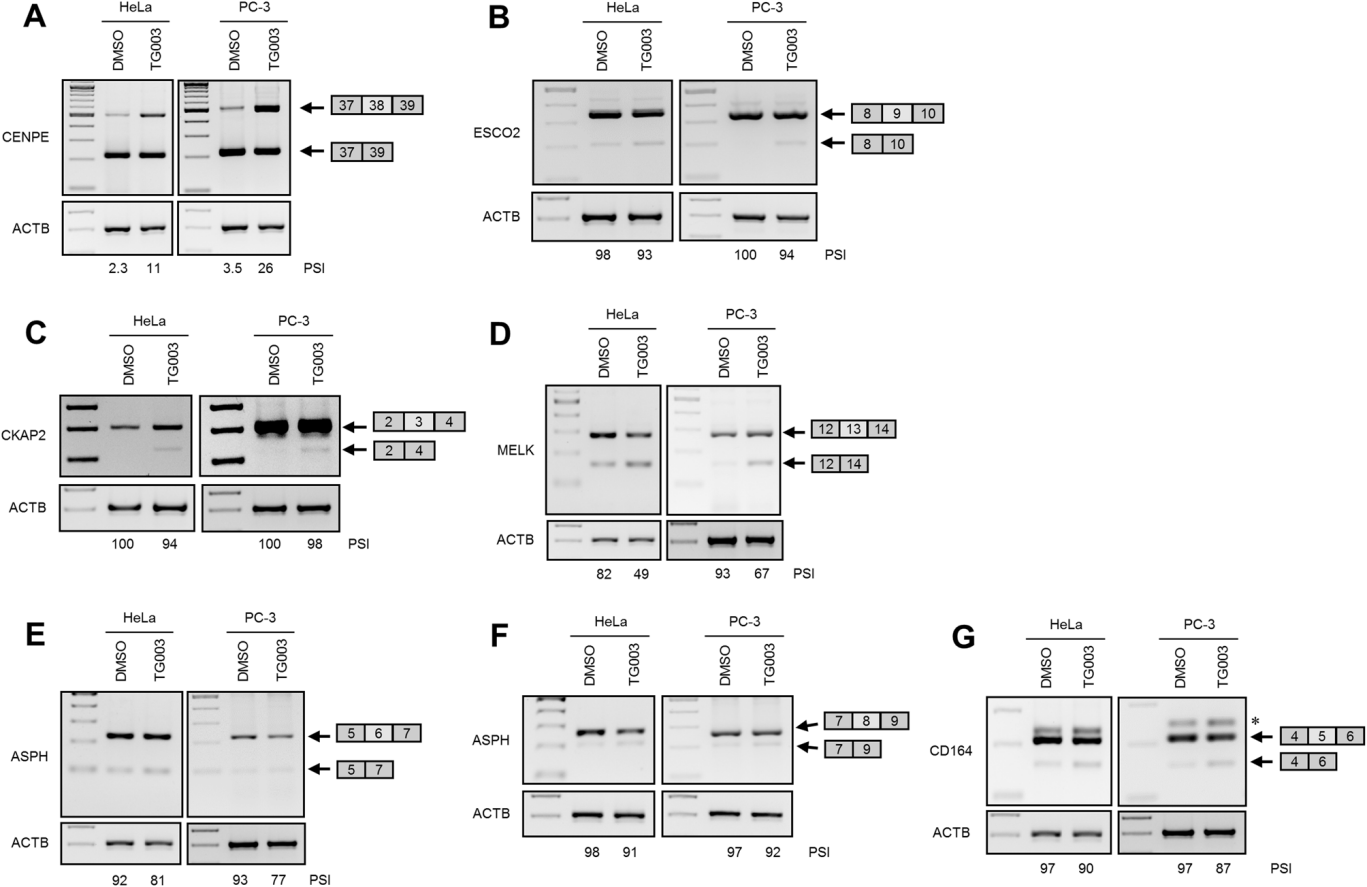
RT-PCR validation for RNA-Seq analysis in HeLa and PC-3 cells. RT-PCR results for (**A**) centromere protein E (CENPE) exon 38; (**B**) establishment of sister chromatid cohesion N-acetyltransferase 2 (ESCO2) exon 9; (**C**) cytoskeleton associated protein 2 (CKAP2) exon 3; (**D**) maternal embryonic leucine zipper kinase (MELK) exon 13; (**E**) aspartate beta-hydroxylase (ASPH) exon 6 and (**F**) exon 8; and (**G**) CD164 exon 5. β-actin (ACTB) served as a loading. PSI, percent spliced-in. *, a stable hybrid of two splice products (exon 5 inclusion and skipping products) was confirmed by TA-cloning in (**G)**.

To understand the mechanisms that underpin growth suppression and apoptotic induction following TG003 treatment in PC3 cells, we searched for TG003-targeted alternative splice events. By analyzing the RNA-Seq datasets for previously TG003-treated HeLa cells^32^, we identified 332 exon-skipping events and 286 exon-inclusion events as TG003 target events (Table 1). The most frequent alternative splicing events affected by TG003 were skipped exons (supplementary Figure S2A). Among 332 exon-skipping events, 270 events occurred in exons for productive forms of mRNAs, leading to in-frame deletion or production of truncated protein due to a premature termination codon (Fig. 6). In concordance with growth suppressive effects obtained through TG003 treatments, gene ontology (GO) analysis of TG003-responsive alternative splicing events revealed that cell division (GO:0051301), cell cycle (R-HAS-1640170), and DNA replication (GO:0006260) were among the most enriched terms (supplementary Figure S2B; for a comprehensive list, see Supplementary Figure S3). We subsequently validated the RNA-Seq analysis results by RT-PCR in HeLa and PC3 cells, for genes previously reported as oncogenic or malignancy factors, including centromere protein E (CENPE), establishment of sister chromatid cohesion N-acetyltransferase 2 (ESCO2), cytoskeleton associated protein 2 (CKAP2), maternal embryonic leucine zipper kinase (MELK), aspartate beta-hydroxylase (ASPH), and CD164, transmembrane cell adhesion molecule (Fig. 7). All of the exons we examined showed consistent changes confirming the RNA-Seq analysis results in both HeLa and PC3 cells, with 7/7 in HeLa and 5/7 in PC3 cells exhibiting ≥ 5.0 of ΔPSI (Fig. 7), indicating that TG003-targeted splice events are largely conserved between HeLa and PC3 cells.

**Table 1:** List of validated TG003-responsive alternative splice events ^32^.

Gene symbol	Gene name	TG003-dependent event	Event ID	Validation (ΔPSI) HeLa cells	Validation (ΔPSI) PC3 cells
*CENPE*	Centromere protein E	Inclusion, exon 38	25664	Success (8.9)	Success (22.7)
*ESCO2*	Establishment of sister chromatid cohesion *N*-acetyltransferase 2	Skipping, exon 9	41800	Success (4.4)	Success (5.6)
*CKAP2*	Cytoskeleton associated protein 2	Skipping, exon 3	82737	Success (5.9)	Success (1.9)
*MELK*	Maternal embryonic leucine zipper kinase	Skipping, exon 13	83190	Success (33.3)	Success (25.8)
*ASPH*	Aspartate beta-hydroxylase	Skipping, exon 6	2109	Success (11.5)	Success (15.2)
*ASPH*	Aspartate beta-hydroxylase	Skipping, exon 8	2108	Success (6.5)	Success (4.0)
*CD164*	CD164 molecule	Skipping, exon 5	82773	Success (6.9)	Success (10.2)

The seven top hits are shown, listing the gene symbols and names, the nature of the altered splicing event, the event ID, and the validation of the ΔPSI in both HeLa and PC3 cells.

## Discussion

Interest in the CDC2-like (CLK) splice factor kinases has grown steadily as they are increasingly implicated in a wide range of developmental and pathological processes including prostate cancer. Our previous work on the splice factor kinase SRPK1 in the prostate cancer context demonstrated that its inhibition may set the scene for novel treatments^41^. We found that the inhibition of SRPK1 exhibits a potent anti-angiogenic effect by reducing the expression of the pro-angiogenic splice isoform of VEGF-A, whose expression is increased by SRSF1, an oncogenic splice factor^42^ and SRPK1 and CLK1 substrate. We therefore decided to investigate the potential of targeting CLKs in prostate cancer cells. We selected the benzothiazole TG003, a readily available and well-established CLK1 and CLK4 inhibitor^31^. We applied it to two well-studied prostate cancer cell lines, PC3 and DU145; and for comparison, normal prostate epithelium (the PNT2 cell line).

We found that TG003 convincingly reduced cell proliferation. Consistent with a previous report that CLK splice factor kinase inhibition can induce apoptosis^39^, we observed a clear increase in apoptosis by TG003 in all three cell lines. We then examined the effect of TG003 on cell biology. TG003 strongly suppressed their growth following bi-weekly injections of TG003 (that result in an approximate concentration of 50 µM in the mouse). We observed an increase in E-cadherin and a decrease in vimentin expression consistent with a reversal of EMT. Having observed the effect of TG003 on prostate cancer cells in vitro, we performed a xenograft experiment. The PC3 xenografts grew rapidly; however, TG003 strongly suppressed their growth following bi-weekly injections of 50 µM TG003. The mice did not appear adversely affected by multiple TG003 treatments.

Given these findings we performed an RNASeq analysis to understand the pathways through which TG003 might be exerting its potent effect on prostate cancer cell growth. We exploited a dataset obtained from a previous RNASeq analysis of TG003-treated HeLa cells^32^ and identified 332 exon-skipping and 286 exon-inclusion events. We focused on a set of genes previously reported to be associated with oncogenic or malignant processes. CENPE plays a major role in chromosome segregation in M-phase, and TG003 promotes inclusion of exon 38, resulting in reduced protein expression^33^. RT-PCR confirmed this splicing change in treated HeLa and PC3 cells. ESCO2 is a cohesion regulator that contributes to cancer cell proliferation^43^. We confirmed induced skipping of exon 9 that results in in-frame deletion of 49 amino acids at the carboxy-terminal. CKAP2 is involved in ovarian cancer tumorigenesis through FAK-ERK pathway and a potential prognostic marker of HER2-negative luminal type breast cancer^44^. TG003 treatment induced skipping of exon 3 introducing a premature termination codon. MELK (maternal embryonic leucine zipper kinase) is an oncogene that is upregulated in high-grade prostate cancer^45^. TG003 induced exon 13 skipping removing 41 critical amino acids. ASPH is reported as a malignant factor in hepatocellular carcinoma, glioma, and pancreatic cancer^46–48^, and its targeting reduces proliferation and invasion of prostate cancer cells through Notch signalling modulation^49^. RT-PCR confirmed that TG003 induced skipping of exon 6 and 8, resulting in in-frame deletion of 44 and 20 amino acids, respectively, within the ASPH amino-terminal region. The sialomucin CD164 promotes Akt/mTOR signaling and known as a malignant factor in various cancers^50^. TG003 promoted skipping of exon 5 to induce in-frame deletion of 20 amino acids. Collectively, our results demonstrate that TG003 treatment induces deletion or truncation of oncogenic and malignancy-associated gene products by impairing their alternative splicing regulation.

There is currently a significant worldwide effort to develop specific, highly and pharmacologically effective inhibitors of oncogenic splice factor kinases including the CLKs^51–54^. In summary, we show that the benzothiazole CLK inhibitor TG003 potently inhibits prostate cancer cell growth in vitro and in vivo*.* TG003 reduces cell proliferation, increases apoptosis, reverses EMT marker expression and reduces cell invasion. Further research is now needed to develop clinically viable CLK inhibitors.

## Supplementary Information

Supplementary Figure S1 & S2

Supplementary Figure S3
