# Supplementary material for: CDC2-like (CLK) protein kinase inhibition as a novel targeted therapeutic strategy in prostate cancer
Source: Sci Rep. 2021 Apr 12;11:7963. doi: 10.1038/s41598-021-86908-6 (PMC8041776; doi:10.1038/s41598-021-86908-6)

SUPPLEMENTARY INFORMATION

CDC2-like (CLK) protein kinase inhibition as a novel targeted therapeutic strategy in prostate cancer

Simon Uzor, Sean R. Porazinski, Ling Li, Bethany Clark, Masahiko Ajiro, Kei Iida, Masatoshi Hagiwara, Abdullah A. Alqasem, Claire M. Perks, Ian D. Wilson, Sebastian Oltean Michael R. Ladomery

Supplementary Figure S1. (A) Treatment of PC3 cells for 48hrs with TG003 reduces phosphorylation of SR protein splice factors. The western blot was performed using the primary antibody mAb104 which detects phosphorylated epitopes on SR proteins. **(B)** Confirmation of CLK1 over-expression in PC3 cells stably transfected with the pAM92 human CLK1-expressing plasmid (CLK1 western blot above, and corresponding total protein on the same PVDF membrane below). The yellow asterisk indicates the position of full-length CLK1.

Supplementary Figure S2. An RNASeq analysis was performed on HeLa cells treated with 50μM TG003 for 48 hours. **(A)** Number of alternative splicing events detected in TG003-treated cells. SE: skipped exons; MXEB: mutually excluded exons; A5SS: alternative 5' splice sites; A3SS: alternative 3' splice sites; RI: retained introns. **(B)** Gene Ontology (GO) analysis of the TG003-targeted splice events in HeLa cells. The graph illustrates the result of a gene ontology analysis.

Supplementary Figure S3. Excel list of alternative splicing events that are affected by TG003 treatment of HeLa cells.

Supplementary Figure S1

A

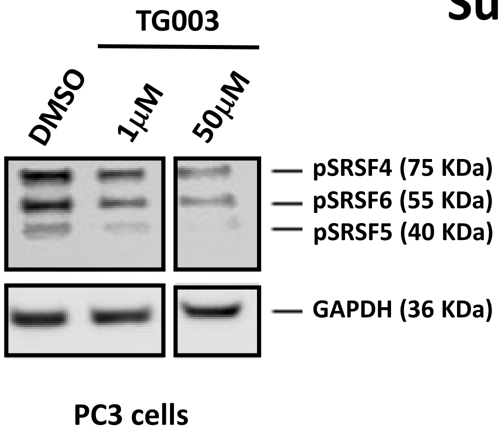

B

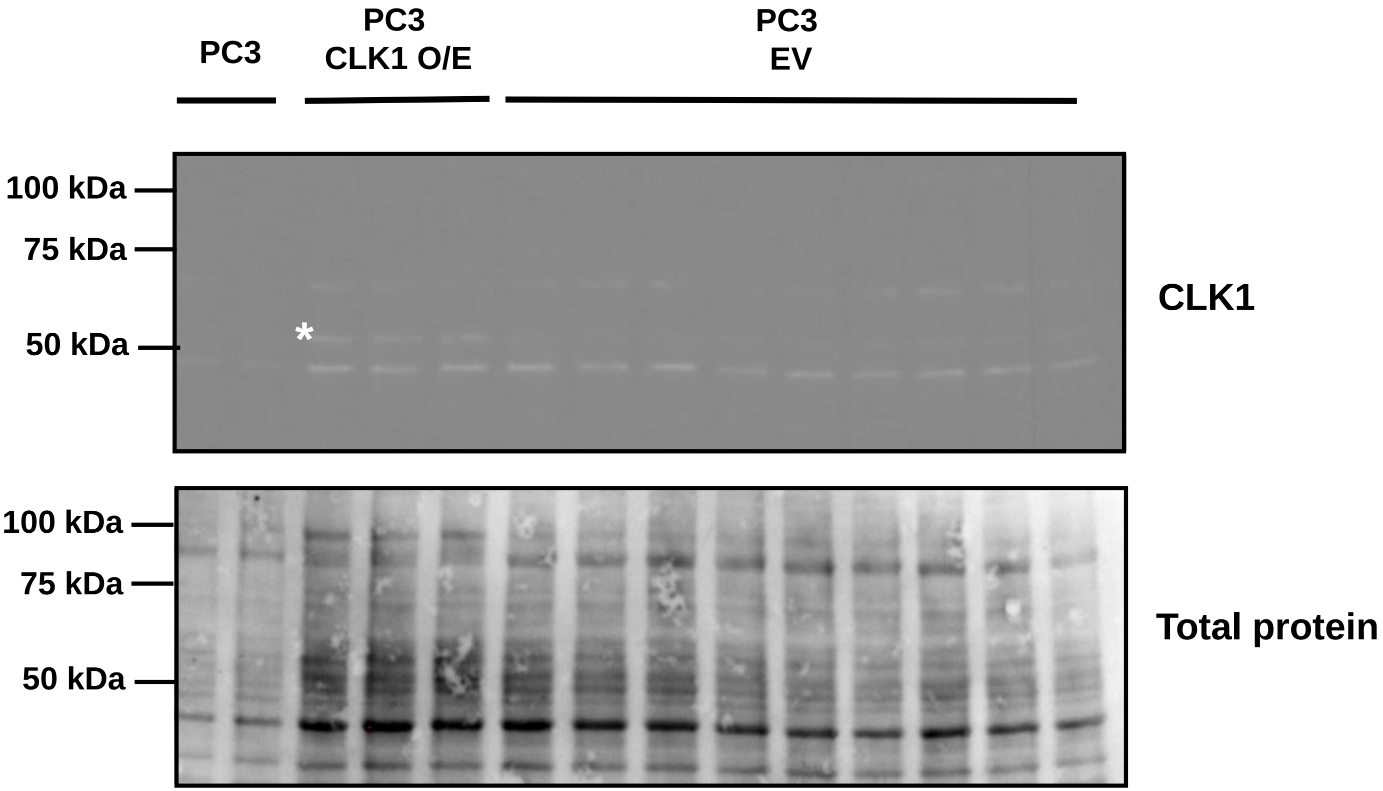

Supplementary Figure S2

A

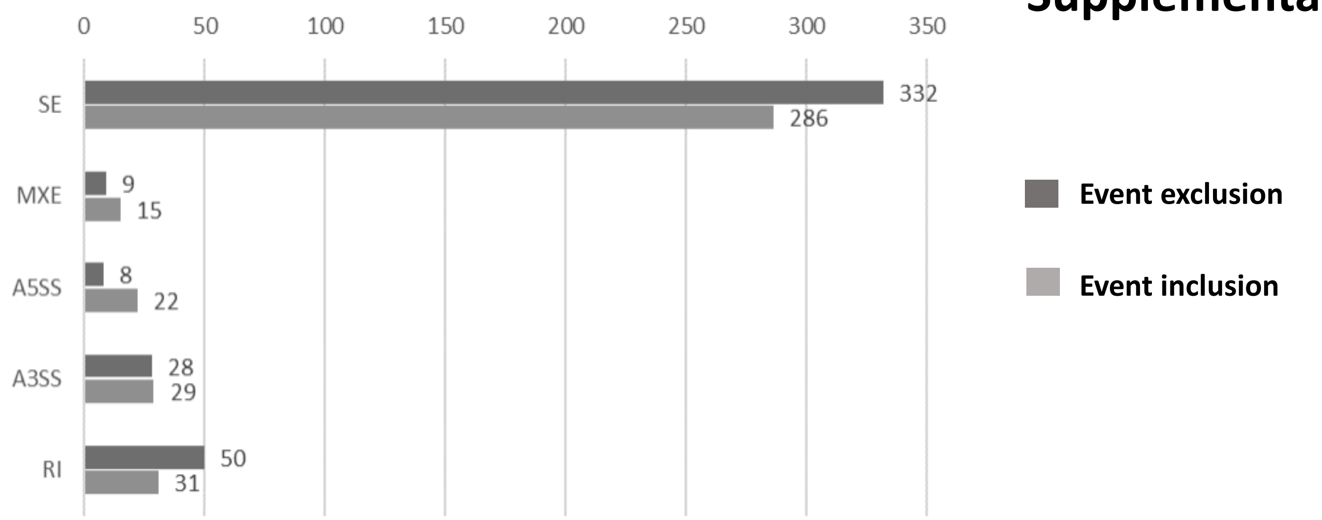

B

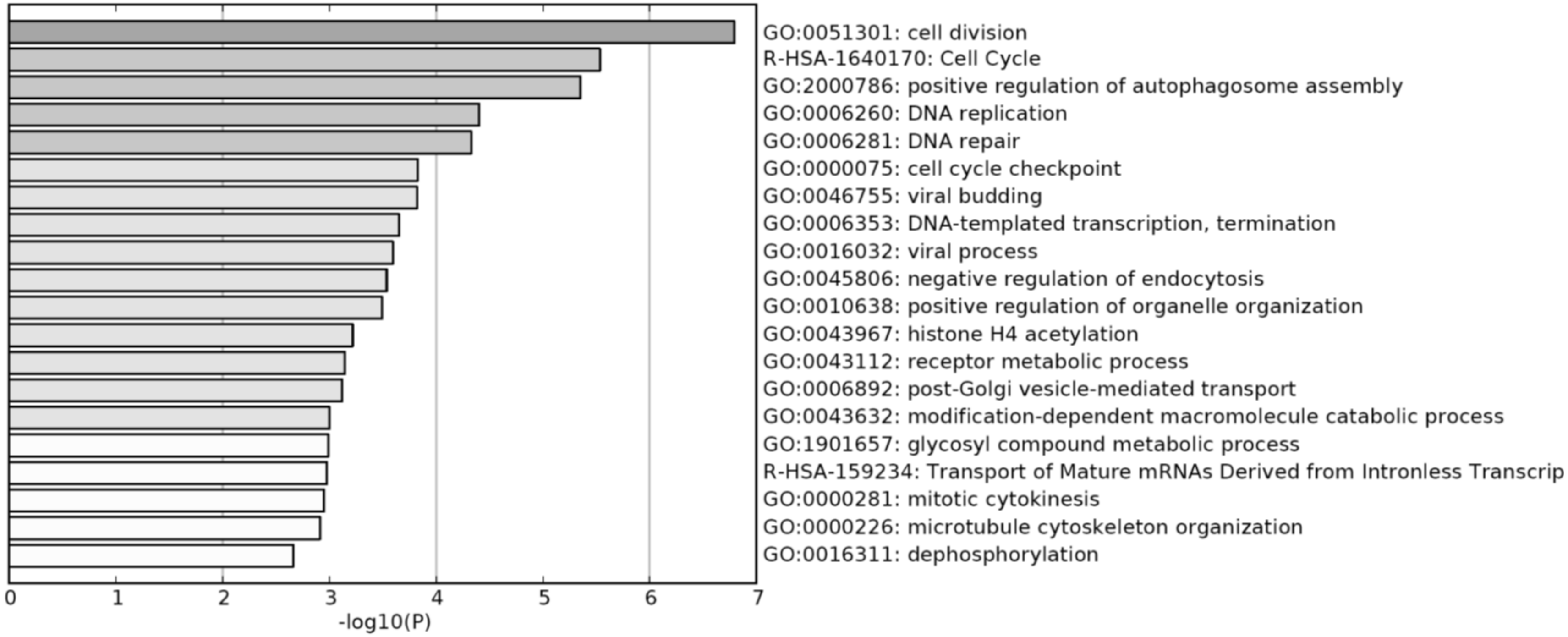

Supplement: Supplementary file 1 — Supplementary Figure S1 & S2 [file 41598_2021_86908_MOESM1_ESM.pdf]
